# Knowledge and experience with cochrane and evidence based medicine among health professionals in Debreberhan Referral Hospital in Ethiopia: a cross-sectional survey

**DOI:** 10.11604/pamj.2018.30.162.14667

**Published:** 2018-06-22

**Authors:** Omar Abdulwadud, Fiseha Tadesse, Getachew Yilma, Metti Midekssa, Irina Ibraghimova

**Affiliations:** 1 American International Health Alliance, HIV/AIDS Twinning Centre, Addis Ababa, Ethiopia; 2 DebreBerhan Referral Hospital, Addis Ababa, Ethiopia; 3 Argo Consulting, Zagreb, Croatia

**Keywords:** Survey, cochrane, cochrane South Africa, evidence based medicine, health professionals, Ethiopia

## Abstract

**Introduction:**

Cochrane generates and disseminates high-quality systematic reviews through the cochrane library. We surveyed Ethiopian health professionals' knowledge and experience with cochrane, the cochrane library and Evidence Based Medicine (EBM).

**Methods:**

A cross-sectional survey was conducted using a convenient sample of health professionals in DebreBerhan Referral hospital in Ethiopia. Participants completed a pre-tested self-administered survey before EBM training. Data were analyzed using Fisher's exact or Chi-Squared test with Yates' correction. The strength of association between variables was quantified using odds ratios with 95% confidence intervals.

**Results:**

The response rate was 71.4% (35/49). Over half (54.3%) of the sample were males; 68.6% aged ≤ 30 years; 54.3% were physicians and 37.1% were nurses. Up to 65.7% had heard about Cochrane and only two knew cochrane South Africa as their reference centre. Nearly 48.6% were aware of the cochrane library, of whom 46% accessed it however; none used it for lacking awareness, search skills, access to internet and time constraints. Majority had a positive attitude towards EBM; 45.7% had heard of EBM; 74.3% rated their EBM knowledge as low; 74.3% lacked EBM training; and 88.6% were keen to attend EBM course. Adequate EBM knowledge was correlated with prior training (OR = 3.7, 95% CI 1.9-6.9, P<0.001], high self-assessment of EBM knowledge (OR = 0.27, 95% CI 0.14-0.51, P<0.001), male gender (P = 0.04), a positive attitude towards EBM (P = 0.001) and awareness of Cochrane (P = 0.004).

**Conclusion:**

Ethiopian health professionals have unmet training needs and want support through professional development workshops and an improved education system to provide high-quality evidence-based healthcare.

## Introduction

Evidence Based Medicine (EBM) is the integration of clinical expertise with the best research evidence and patient values and preferences to formulate the right decisions to care for individual patients [1]. The EBM paradigm surfaced in the early 1990's to guide medical decisions [2]. Since then, Cochrane has been transforming the way health decisions are made globally [3] by generating, maintaining and disseminating systematic reviews in the Cochrane Library, which are relevant, reliable and inform a wide ranging people including health professionals [4]. The reviews are accessible free of charge in low-income countries [5] and can inform a wide-ranging people including health professionals (HPs). Applying evidence in clinical practice makes treatment cost effective and enhances healthcare quality by bridging the gap between best practice and usual care and preventing harmful and unbeneficial practices [6, 7]. This is why EBM competency applies to all HPs, thus EBM training is essential [7, 8]. In Africa, however, practicing HPs face several challenges to learn and practice EBM. Basically, EBM teaching is lacking in medical curriculum and few institutions offer EBM courses outside South Africa [9-11]. The health system is also unfavourable for EBM, particularly the health infrastructure, access to internet, online EBM resources together with high workload and time constraints of HPs [12-18]. The political commitment and national policy to support EBM have not changed either [7]. For example, no EBM experts at the Federal Ministry of Health in Ethiopia to synthesize evidence and advice policy-makers on major public health program such as maternal and newborn health [19]. Established in 1997, Cochrane South Africa is one of the 14 Regional Cochrane Centres that promotes Cochrane activities in Africa and serves as a reference centre for 25 countries, including Ethiopia [20]. Although the number of Cochrane authors and systematic reviews from the region has increased in the past two decades [21], awareness of Cochrane, Cochrane South Africa and use of the Cochrane systematic reviews is still poor [13-16, 22-24]. Little is also known about EBM diffusion into health and education sectors in countries such as Ethiopia. To bridge this gap, we conducted a survey of Ethiopian health professionals to assess their awareness and experience about Cochrane, the Cochrane Library and EBM.

## Methods

**Study setting and design**: A cross-sectional survey was conducted in September 2014 at DebreBerhan Hospital in Ethiopia. It is the only public regional referral hospital in the area serving about 2.4 million population. The hospital has 130 beds, 366 employees (261 technical, 105 administrative), treats about 450 patients daily and provides curative and preventive services at inpatient and outpatient departments [25].

**Participants**: They were mainly practicing physicians and nurses who were nominated to participate in EBM training, which was funded by the American International Health Alliance [26]. The trainees were informed about the survey objective, participation was voluntary, assured confidentiality and the survey was approved by the medical director.

**Assessment, collection and data management**: The survey was designed by Cochrane South Africa (CSA) and used in the 2004 study of people in developing countries on the challenges they face to contribute to Cochrane, and has been described previously [22, 24]. After adding five EBM items, the self-administered survey was pretested before data collection. The reliability (internal consistency) of the piloted survey was satisfactory (Cronbach's alpha = 0.78). The survey collected and assessed participants' socio-demographic, knowledge of Cochrane, CSA, EBM, and access and barriers to the Cochrane Library. The 7-items and the scoring method used to asses EBM knowledge were: (1) Heard of EBM (yes = 1, no = 0); (2) Attended EBM training (yes = 1, no = 0); (3) Aware of Cochrane (yes = 1, no = 0); (4) Ethiopian health professionals recognize the Cochrane Library (yes = 1, no=0, don't know=0); (5) EBM knowledge level (none=0, poor = 1, average = 2, good = 3, very good = 4, excellent = 5); (6) Know Cochrane Reference Centre for Ethiopia (CSA=1, don't know=0); and (7) Aware and know to access the Cochrane Library (free access = 1, subscription = 0, don't know = 0). The knowledge score was the total of correct answers. Their attitude towards EBM was measured using 3-items on a three-point scale (yes = 1, no=0, don't know = 0). The questions were: (1) EBM is useful in clinical practice; (2) My hospital promotes EBM practice; and (3) I am interested in EBM course. The overall attitude score was the total number of correct answers. The survey also included one-closed and two open-ended items to assess participants' awareness of the Cochrane Library, which is freely available at the point of care in Ethiopia, and their barriers to and enablers of accessing the Cochrane Library.

**Data analysis**: The Shapiro-Wilk test showed the total EBM knowledge scores was normally distributed (Statistic 0.965, df=35, P = 0.313) but not the total attitude scores (Statistic 0.795, df = 35, P<0.001). Using the mean value of 4.26, the participants' total knowledge score was classified as inadequate or adequate. To categorize their overall attitude towards EBM score as negative or positive, the median score of 2 was applied. The association between categorical parameters were tested by Fisher's exact or Chi-Squared tests with Yates' correction. An odds ratio with 95% confidence interval was calculated to assess the strength of association. All statistical tests were two tailed and a P value of less than 0.05 was applied for statistical significance. The IBM SPSS Statistics for Windows (Version 20.0) was used for data analysis.

## Results

Participants: Out of 49 participants who attended EBM training, 35 (71.4%) completed the survey. The median time to complete the survey was 11 min (Interquartile range 9-20). Sixteen (46%) of the participants were females and 19 (54.3%) were males; 24 (69%) were ≤ 30 years old and 11 (31.4%) between 31-40 years of age; 19 (54%) were physicians, 13 (37%) nurses, and 3 (9%) were in other profession (2 pharmacists, 1 IT person). Of the total 19 physicians, 15 (79%) were general practitioners and four (21%) specialists (dentist, internist, paediatrician, psychiatrist). All were very comfortable or comfortable in reading and writing English language. The study participants (n = 35) and nonparticipants (n = 14) were comparable on a range of socio-demographic variables. For easy of analysis and clarity, the responses from three other professionals and 13 nurses was combined and presented in this report.

**Figure 1:**
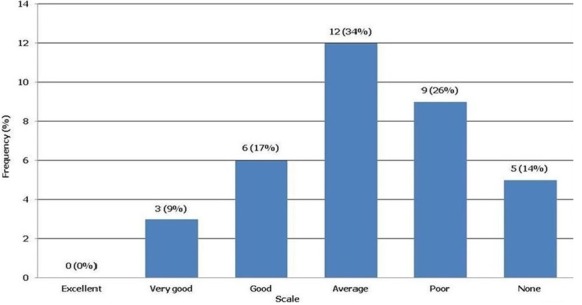
Respondents’ self-assessment of evidence based medicine knowledge level, Ethiopia, 2014

**Knowledge of Cochrane and evidence based medicine**: Twenty-three (65.7%) of the participants have heard about Cochrane and 12 (34.3%) did not. Awareness of Cochrane was 68.8% for nurses and 63.2% for physicians with no significant difference between the two groups (P = 0.73). Most gained their awareness of Cochrane from conferences or internet sites (43.5%), followed by colleagues (39%) and other sources (17.4%) such as journal articles, supervisors and training. Overall awareness of Cochrane was not associated with gender (P = 0.14). Asked whether Ethiopian health professionals were familiar with Cochrane, 19 (54.3%) stated that they were. When asked to identify the Cochrane Reference Centre for Ethiopia, only two (5.7%) participants identified Cochrane South Africa and the large majority (n = 33, 94.3%) did not know. Of the total participants, 16 (45.7%) had heard of EBM and 19 (54.3%) had not. Awareness of EBM among physicians (68.4%) was significantly higher than nurses (18.8%) (Chi-Squared = 6.750, df = 1, P = 0.009). Similarly, significantly more male participants (68.4%) than females (19%) had awareness of EBM (Chi-Squared = 6.75, df = 1, P = 0.009). Awareness of EBM was not associated with age group (P = 0.26). When asked if they had attended EBM training, only 9 (25.7%) indicated that they had and 26 (74.3%) had not attended any training. The level of EBM training was comparable between nurses (31.3%) and physicians (21.3%) (P = 0.07). Figure 1 shows the participants' self-assessment of EBM knowledge using a six-point scale. Nobody evaluated their EBM knowledge level as excellent. About 69% of nurses and 79% of physicians believed their EBM knowledge was low, with no significant difference between the two professions (P = 0.15). Generally, participants assessment of EBM knowledge level was not related to their age group (P = 0.57) or gender (P = 0.39).

**Attitude towards evidence based medicine**: The large majority approved that the use of EBM in clinical practice was beneficial (**Table 1**). Most of the nurses (75.0%) and physicians (84.2%) also recognized such benefits (p = 0.67). The follow-up question asked whether or not their hospital promotes EBM in clinical practice. Overall, less than half stated that it was promoted. When asked about their interest to attend EBM training, a high percentage of them expressed interest. Despite nurses (94%) showing slightly more interest in training than physicians (84%), the difference was not significant (P = 0.60). There was no association between participants willingness to attend EBM training and their age group or gender.

**Table 1:** Respondents’ attitude towards evidence based medicine, Ethiopia, 2014

Item	Yes No (%)	No No (%)	Don’t know No (%)
The use of EBM in clinical practice is beneficial	28 (80.0)	3 (8.6)	4 (11.4)
My hospital promotes EBM practice	15 (42.9)	13 (37.1)	7 (20.0)
I am interested in EBM training course	31 (88.5)	3 (8.6)	1 (2.9)

EBM = Evidence Based Medicine

**Factors associated with knowledge of evidence based medicine**: Using the criterions described in the data analysis section, 16 (45.7%) of the participants were judged to have adequate knowledge and 19 (54.3%) inadequate knowledge of EBM. The rate of adequate knowledge of EBM was comparable between nurses (43.8%) and physicians (47.4%) (P = 0.83). Similarly, 13 (37.1%) were considered having a positive attitude and 22 (62.9%) a negative attitude towards EBM. The proportion of nurses (25%) and physicians (47%) with a positive attitude was comparable (P = 0.31). The participants with adequate knowledge of EBM were more likely to show a positive attitude towards EBM (P = 0.001) (Table 2). Adequate knowledge of EBM among participants with prior training was significantly higher than those without training (P<0.001). Adequate knowledge was 56.3% for participants who assessed their EBM knowledge as high and 43.7% for those who measured it low (P<0.001). The participants with awareness of Cochrane were more likely to have adequate knowledge compared to those lacking awareness (P = 0.004). Male participants were slightly more likely to have adequate knowledge than female participants (P = 0.04). There was no association between participants' adequate knowledge of EBM and their profession, ability to read or write English, or age group (Table 2).

**Table 2:** Knowledge of evidence based medicine in relation to study parameters, Ethiopia, 2014

Characteristics	Total No (%)	Knowledge status		Odds ratio (95% CI)	P value
		Adequate No (%)	Inadequate (No (%)		
***Age group (Years)***					
≤ 30	24 (68.6)	9 (56.3)	15 (78.9)	0.34 (0.08‒1.51)	0.28
31‒40	11 (31.4)	7 (43.7)	4 (21.1)		
***Sex***					
Male	19 (54.3)	12 (75.0)	7 (36.8)	5.14 (1.18‒22.28)	0.04
Female	16 (45.7)	4 (25.0)	12 (63.2)		
***Speciality/Profession***					
Nurse	16 (45.7)	7 (43.7)	9 (47.4)	0.86 (0.23‒3.29)	0.74
Physician	19 (54.3)	9 (56.3)	10 (52.6)		
***Ability to read English***					
Very comfortable	17 (48.6)	8 (50.0)	9 (47.4)	1.11 (0.29‒4.21)	0.75
Comfortable	18 (51.4)	8 (50.0)	10 (52.6)		
***Ability to write English***					
Very comfortable	15 (42.9)	8 (50.0)	7 (36.8)	1.71 (0.44‒6.63)	0.66
Comfortable	20 (57.1)	8 (50.0)	12 (63.2)		
***Attended EBM training***					
Yes	9 (25.7)	9 (56.3)	0 (0)	3.71 (1.97–6.99)	<0.001
No	26 (74.3)	7 (43.7)	19 (100)		
***Self-assessment of EBM knowledge level***					
High*	9 (25.7)	9 (56.3)	0 (0)	0.27 (0.14–0.51)	<0.001
Low‡	26 (74.3)	7 (43.7)	19 (100)		
***Aware of Cochrane***					
Yes	23 (65.7)	15 (93.7)	8 (42.1)	20.63 (2.24–189.84)	0.004
No	12 (34.3)	1 (6.3)	11 (57.9)		
***Attitude towards EBM†***					
Positive	13 (37.1)	11 (68.8)	2 (10.5)	18.70 (3.07‒113.89)	0.001
Negative	22 (62.9)	5 (31.2)	17 (89.5)		

† Classification criteria described in data analysis section CI= Confidence interval EBM=Evidence Based Medicine

* Health Professionals who assessed their knowledge as ‘’very good’’ and ‘’good’’

‡ Health Professionals who assessed their knowledge level as ‘’average’’, ‘’poor’’ and ‘’illiterate’’

**Knowledge of the Cochrane library**: Of the total 35 participants, 17 (48.6%) reported awareness of the Cochrane Library and knew it was free online access in Ethiopia. Yet, 18 (51.4%) were unaware and had no idea how to access it. This result did not differ between nurses (50%) and physicians (47.4%). A follow-up question asked if the general population of Ethiopian health professionals had awareness of the Cochrane Library. Fifteen (42.9%) of the participants believed that they were aware whereas 20 (57.1%) were unsure.

**Use and factors associated with accessing the Cochrane library**: Twenty-four (68.6%) of the participants had accessed the Cochrane Library while 11 (31.4%) did not. None of the 24 who indicated accessing the resource had used it because of difficulties. Their two most common individual barriers were unawareness of the resource (54.2%) and lack of search skills (45.8%). They also reported system barriers involving the lack of access to reliable internet (45.8%), inadequate facilities or resources relating to computers and library services (12.5%), poor access to the Cochrane Library and insufficient time at work (8.3%).The study participants also proposed enablers to overcome their barriers. The majority (80%) wanted hands-on training to develop information searching skills; 45.7% suggested raising awareness of the Cochrane Library; 14.3% proposed easy access to internet; and 8.6% sought more computers to be accessible at the point of care.

## Discussion

In the present survey, most health professionals have heard of Cochrane from various sources. This finding is consistent with evidence from India [27] but lower than other surveys [22, 23]. The discrepancies could be attributed to the differences in response rates, recruitment strategies and methods across the surveys. The majority, however, did not identify Cochrane South Africa (CSA) as their reference centre. An organized strategy is required to better promote CSA and Cochrane activities in Ethiopia. Essentially, future strategies must focus on academics, research centres and higher educational institutions in the country. The large majority in this survey showed a welcoming attitude towards EBM, which is consistent with the literature [12, 16, 28-31].Their overall attitude towards EBM was also significantly associated with their general knowledge of EBM. While the findings are encouraging and imply they would support EBM practice, one must be cautious as it was based on self-assessment and may not translate into good clinical behaviour. In the current survey, the majority did not have awareness of, knowledge and training in EBM. These findings are consistent with previous studies [12-15, 28-32] and indicate that our participants have not been exposed to EBM education. This conclusion is based on the evidence that 69% of them were young (≤ 30 years) and graduated not long ago. Overall, our findings highlight the underlying weakness in the medical curriculum in Ethiopia. The most effective intervention to address these intractable barriers to EBM practice is by integrating EBM education into medical (health) education core curriculum and continuing education programs [8]. The use of evidence-based resource helps to inform clinical practice. In Ethiopia, although the Cochrane Library is free, less than half were aware of the resource. Strikingly, none of those who accessed it have used it in practice either. This is not surprising given similar observations have been reported before [30-34]. In Nigeria, doctors' use of the Cochrane Library was reported as 10% [13] and 7% [14].

Equally, 8.5% of Sri Lankan doctors [32] and 4.3% of Jordanian family doctors [34] use it. Overall, these usage rates are low and a better intervention is warranted to enhance awareness and use of the Cochrane Library in developing countries. In Ethiopia, it is vital to develop a partnership between key local stakeholders, consisting of academics, teaching hospitals, professional organizations and the 25 medical schools [35], to institute a framework for better promotion strategy. The partnership can also help to generate a critical mass of cohorts able to use the Cochrane Library in the long-term. In Sub-Saharan Africa, the barriers to access online evidence-based resources have been reported [12-18]. Our participants also identified barriers to access the Cochrane Library compatible with previous studies. Their lack of awareness regarding the resource requires awareness raising at staff meeting, clinical rounds and journal clubs. To develop their information searching skills, providing hands-on-training as part of professional development program remains necessary. By downloading the Cochrane Library tutorial for free from Cochrane website, clinical staff can also develop their search skills. Efficient infrastructure, reliable access to internet and online EBM resources are central to access online evidence [33]. Yet, our participants identified structural and resource related barriers to access the Cochrane Library. So, it is necessary to invest adequate funding to upgrade the hospital infrastructure and make computers more accessible for staff to retrieve evidence at the point of patient care. The survey strength was that it achieved a high response rate (71.4%) comparable to other studies with similar population in Sub-Saharan Africa [14, 30]. Our sample size was small but it represented 12% of the total clinical staff in the study hospital. The study participants and non-participants were compared and found to be similar. We also collected data using a pretested survey and conducted the research as part of a training program without additional budget or resources. Despite generating useful baseline data, the survey has limitations. Because of the small self-selected convenient sample, the findings are not generalizable to all health professionals or other settings. The use of a cross-sectional study design also limits data relevance to a single point of time. Our results also depended on participants' self-assessment of outcome measures that might be biased. The associations between EBM knowledge and other study factors were based on a bivariate analysis, and the relationship could be from the influence of unsuspected or uncontrolled confounders. The wider 95% confidence interval for some of the odds ratios also decreases precision and reflects the small sample size.

## Conclusion

Ethiopian practicing health professionals have unmet training needs to provide evidence-based healthcare. A focused strategic plan of action should be developed to overcome their personal and organizational barriers to practice EBM. Establishing a hospital committee that develops a framework and policy for EBM program must be considered. Awareness sensitization campaign, in-service training and upgrading the health infrastructure, including the internet service are critical to promote the use of Cochrane Library among clinical staff. The set up of EBM journal club in hospitals also helps an increased awareness and use of evidence, as well as assist clinical staff to keep up-to-date with the medical literature.

### What is known about this topic

- Major barriers to awareness of Cochrane, the Cochrane Library and evidence-based practice exist in Africa;
- Strong political commitment, leadership and policy are lacking for evidence-based practice in Africa.

### What this study adds

- Medical (health) institutions in Africa can have positive impact in fostering awareness about Evidence Based Medicine and use of the Cochrane Library among future cohorts of health workers;
- The importance of integrating Evidence Based Medicine into continuing professional development program and medical (health) education in Africa.

## Competing interests

The authors declare no competing interest.
